# First impressions of a new face are shaped by infection concerns

**DOI:** 10.1093/emph/eoad025

**Published:** 2023-08-07

**Authors:** Paola Bressan

**Affiliations:** Department of General Psychology, University of Padova, Padova, Italy

**Keywords:** behavioral immune system, face perception, first impressions, pathogen avoidance, disgust, sickness communication

## Abstract

Along with a classical immune system, we have evolved a behavioral one that directs us away from potentially contagious individuals. Here I show, using publicly available cross-cultural data, that this adaptation is so fundamental that our first impressions of a male stranger are largely driven by the perceived health of his face. Positive (likeable, capable, intelligent, trustworthy) and negative (unfriendly, ignorant, lazy) first impressions are affected by facial health in adaptively different ways, inconsistent with a mere halo effect; they are also modulated by one’s current state of health and inclination to feel disgusted by pathogens. These findings, which replicated across two countries as different as the USA and India, suggest that instinctive perceptions of badness and goodness from faces are not two sides of the same coin but reflect the (nonsymmetrical) expected costs and benefits of interaction. Apparently, pathogens run the show—and first impressions come second. **Lay Summary:** Our first impressions of strangers (whether they seem trustworthy, intelligent, unfriendly, or aggressive) are shaped by how healthy their faces look and by our unconscious motivation to avoid infections. Bad and good impressions turn out to reflect the concrete, potentially vital, expected costs and benefits of interacting with our fellow humans. Apparently, pathogens run the show—and first impressions come second.

Your face, my Thane, is as a book, where men

May read strange matters.

—Macbeth act 1, sc. 5, l. 61 (1606)

## 1. YOUR FACE IS AS A BOOK

Our forced cohabitation with pathogens has placed an extraordinary selective pressure on our ability to identify infected conspecifics. Thus, we have evolved a suite of automatic responses to signs that, over evolutionary time, were statistically associated with disease. Most such signs show up on the face: pale skin and lips, red eyes, droopy eyelids and mouth corners, a tired and sad expression tend to be interpreted as cues of sickliness [1, 2]. These biases do have a protective function—as disclosed by the finding that we happen to like less the faces of people who, unbeknownst to us, have just received an injection of a toxin rather than of innocuous saline [3].

The primary dimension along which we judge a new face is its ‘goodness/badness’: how much that person looks attractive, intelligent, sociable, responsible—not aggressive, not threatening, not weird, not nasty. These assessments have been shown to be based on the face’s apparent trustworthiness [4]. There are very good reasons for that [5]: ‘neutral’ faces look trustworthy when they naturally resemble happy expressions (U-shaped mouth and inverted-V-shaped eyebrows), and they look untrustworthy when their traits are arranged as though they were communicating negative emotions, such as anger (inverted-U-shaped mouth and V-shaped eyebrows). Expressions reveal intentions, and the intentions of strangers—are they going to befriend us or harm us?—determine how convenient it is to approach or avoid them. In fact, our classification of faces as bad or good is so fast that it even precedes awareness [4].

Yet others’ state of health should be at least as crucial to our fate as their intentions. Bad intentions, taken to the extreme of interpersonal violence, kill worldwide about 400,000 people a year [6]; infections, 15 million [7]. On our choice to seek or eschew contact, thus, whether a person is carrying an infection ought to weigh no less than their mood—arguably, far more. Even people with the happiest disposition and most honorable motives can pass on disease. A touch, a cough, a word, a breath can be enough to kill us [8–10].

In this paper, I test the idea that ostensibly health-unrelated first impressions of strangers (how trustworthy or unfriendly they look) might be pressingly informed by our concern with pathogens. If this is so, our assessment of a new face should be clearly affected by how healthy it appears; and possibly also by our personal instinctive need to avoid infections—as revealed by our own state of health, by our inclination to be disgusted by pathogens,and by whether we live in a high- or low-pathogen country. I examine these points with the help of publicly available data, collected online on 3584 individuals (1969 from India and 1615 from the USA [11]). This dataset features several relevant measures, including people’s current state of health, their propensity to feel disgusted by pathogens, and their first impressions of a new face.

## 2. BAD AND GOOD FIRST IMPRESSIONS ARE NOT TWO SIDES OF THE SAME COIN

Participants saw the photograph of a man with a neutral expression, who was either White (light skin) or Indian (dark skin) and either carried or not a pathogen cue—a severe facial rash added digitally to the image [11]. The study was designed to examine the effects of the man’s ethnicity (same as the participant’s vs. different) and pathogen cue (present vs. absent) on comfort with contact. This was measured as participants’ willingness to shake hands with, or sit next to, the man in the photo.

After expressing their contact comfort with the stranger, participants replied to the question ‘What are your first impressions of this man? Below are several adjectives. Please rate the extent to which you feel that each adjective describes the man in the photo’, on a scale from 0 (not at all characteristic of the man) to 6 (very characteristic of the man). The 14 adjectives, presented in random order, were Dirty, Filthy, Hygienic, Clean, Likeable, Trustworthy, Unfriendly, Aggressive, Capable, Intelligent, Ignorant, Lazy, Rich, and Poor.

Next, for the purpose of checking the validity of the pathogen cue and ethnicity manipulations, participants were asked ‘Does the man look ill or healthy?’, on a scale from –5 (very ill) to +5 (very healthy) and ‘Does this man look like the men in your local community?’, on a scale from 0 (very different from the men in my community) to 10 (very similar to the men in my community). Participants then completed several other measures, including a pathogen disgust sensitivity questionnaire, and reported their current state of health, on a scale from 1 (very good) to 7 (very poor).

The question I examine in this paper is different from the one explored by the original authors, and for this reason I do not focus on the same data. The measures relevant here were used by [11] merely as control variables in their analyses (first-impression ratings and self-reported illness) or as manipulation checks (stranger’s facial health and similarity to locals), and not considered further. In particular, first-impression ratings were not treated as such but used to remove the influence of ‘outgroup stereotypes’: that is, to control for any potential effects, on comfort with contact, of Indian participants’ ‘stereotypes’ about Whites and White participants’ ‘stereotypes’ about Indians.

I performed a principal component analysis with varimax rotation on first-impression ratings, so as to identify the underlying dimensions. Conservatively, I left out those items whose association with perceived health could be expected: clean, hygienic, dirty, filthy, rich, and poor. The analysis run on the remaining eight items revealed two principal components, which were the same in the two cultures and together explained 63% of the variance. These could be interpreted as positive traits, or ‘assets’ (likeable, trustworthy, intelligent, capable) and negative traits, or ‘liabilities’ (ignorant, lazy, unfriendly, aggressive). A score was calculated for each factor, with higher scores corresponding to first impressions of the stranger as presenting, respectively, larger assets and larger liabilities.

## 3. BAD AND GOOD FIRST IMPRESSIONS ARE BOTH SHAPED BY INFECTION CONCERN, BUT IN ADAPTIVELY DIFFERENT WAYS

I tested the prediction that ostensibly health-unrelated first impressions of a new face are shaped by a concern with infectious diseases via two multiple regressions, one for assets and one for liabilities. The independent variables were perceived health, estimated similarity to the men in the local community (which has been shown to increase the face’s perceived health [12]), pathogen disgust sensitivity, self-reported illness, and country.

**Figure 1.**
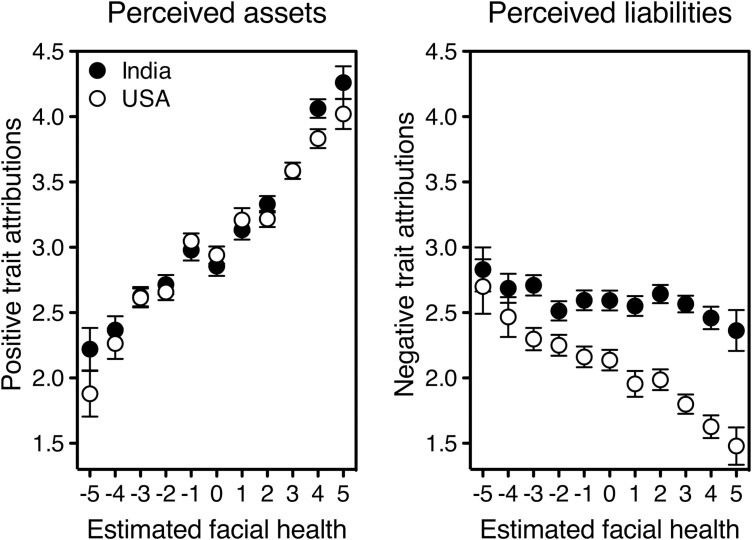
First impressions of health-unrelated assets (left panel) and liabilities (right panel) of a male stranger as a function of his perceived facial health. Data are separately plotted for participants living in India (solid symbols) and the USA (open symbols). All slopes are steeper for assets than for liabilities, suggesting that facial health enhances a stranger’s apparent assets more than it reduces his liabilities (especially in India: in the right panel, the slope is shallower for solid than for open symbols, perceived health × country, beta = 0.12, *P* < 0.0001). Assets (likeable, trustworthy, intelligent, capable) and liabilities (ignorant, lazy, unfriendly, aggressive) were estimated on a scale from 0 to 6. Health was estimated on a scale from –5 (very ill) to +5 (very healthy). Error bars indicate one standard error around the mean.

The stranger’s perceived assets increased with his apparent facial health (beta = 0.39, *P* < 0.0001) and with his similarity to men in the local community (beta = 0.18, *P* < 0.0001), and decreased with participant’s self-reported illness (beta =  –0.054, *P* = 0.0002). Pathogen disgust and country played no significant role (disgust: beta = 0.023, *P* = 0.109; country: beta = –0.016, *P* = 0.287). Importantly, this pattern of results was the same whether or not the face featured a rash, with the only exception that Indians attributed slightly lower assets than did Americans to the stranger with the rash (country: beta = –0.063, *P* = 0.004; see Supplementary Materials for the full analyses and a summary table of all results presented in this paper). The effect of estimated health on positive trait attributions is represented, separately for India and the USA, in Fig. 1, left panel.

The stranger’s perceived liabilities diminished with his apparent facial health (beta = –0.13, *P* < 0.0001) and increased with participant’s pathogen disgust (beta = 0.13, *P* < 0.0001) and self-reported illness (beta = 0.069, *P* < 0.0001). This time, the stranger’s similarity to locals played no role (beta = –0.004, *P* = 0.822) while country did (beta = 0.24, *P* < 0.0001). One wonders whether the effect of perceived health on first impressions could be trivially driven by the participants who were shown the face with the pathogen cue, and might thus possibly have been ‘primed’ by it. This cannot be the case, however, because—just like in the case of perceived assets—the pattern of results was the same whether participants had seen the face with or without the rash (see Supplementary Materials).

The effect of country was due to Indian participants perceiving higher liabilities in a new face than did USA participants. Even though the pattern of significant and nonsignificant results in the two countries was the same, the impact of estimated health on negative attributions was three times larger in the USA (USA, beta = –0.21, *P* < 0.0001; India, beta = –0.07, *P* = 0.003; in Fig. 1, right panel, compare solid and open symbols). In contrast, a healthier-looking face drove up *positive* attributions to an exceedingly similar extent in both countries (Fig. 1, left panel). So, cues of good health may increase the benefits of interacting with a stranger in all environments—but fail to substantially decrease the risks in contexts where the base rate probability that others are inconspicuously harboring an infection is very large, as in India [6]. Note that, across countries, estimated health had a larger effect on assets than on liabilities (USA: *r* = 0.47 vs. *r* = –0.23; India: *r* = 0.45 vs. *r* = –0.06), suggesting again that benefits and costs are not simply opposites of one another. Consistently, a visible pathogen cue weakened positive attributions (*r* = –0.20, *P* < 0.0001) but left negative ones unchanged (*r* = 0.006, *P* = 0.714); the pattern was virtually identical in the USA (–0.19 vs.03) and India (–0.20 vs. –0.02). This asymmetry in the effects of a rash on facial ‘goodness’ and ‘badness’ may indicate that, when confronted with an apparently healthy stranger, it is more adaptive to increase the perceived benefits of contact than to belittle its potential costs.

Of course, the mere fact that perceived health correlates with positive impressions does not imply that it *drives* these impressions; yet there are good reasons to conclude that it does. Sure enough, some faces have more positive neutral expressions than others (say, they look happier due to a naturally upturned mouth). Such faces seem both healthier [13] and more trustworthy [5]. Thus, the association between apparent healthiness and trustworthiness could, in principle, be spurious and entirely mediated by a positive facial expression. However, smiling has been reported to increase a face’s perceived health but not the same face’s perceived trustworthiness [14], which is inconsistent with the interpretation that it is a smiling expression that spuriously links these two attributions. It has in fact been suggested that the very reason why smiles or positive neutral expressions increase perceived health is that they reflect actual health [13]. This goes well beyond the truism that sicker individuals are bound to smile less. Indeed, people who are asked to hold chopsticks in their mouth so as to produce forced smiles show a better cardiovascular recovery from stress [15], and happy people live longer [16].

Comparing the faces with and without a rash supplies an even plainer argument against the idea that the driving force here is not apparent healthiness but some other unmeasured, allegedly disease-unrelated trait that happens to affect both apparent healthiness and likeability—such as a cheerful countenance, a relaxed expression, or attractive lineaments. In the absence of a rash, seeing the stranger as sicker rather than healthier lowered his apparent assets in much the same way as did seeing a pathogen cue on his face. And yet, the rash’s influence could not possibly be mediated by some unspecified confound, as the photos were exactly the same and the rash had been photoshopped on them. That a perceived rash can obviously bias first impressions, while the effects of perceived sickliness must be an artifact and actually produced by some other better variable, does rather stretch the imagination.

**Figure 2.**
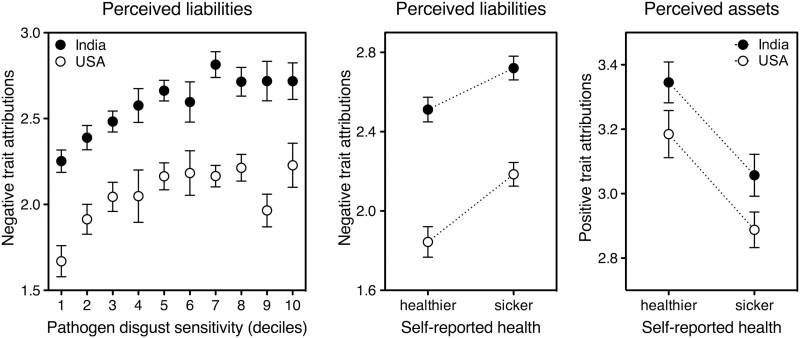
People’s motivation to avoid infections affects their first impressions of a new face. Data are separately plotted for participants living in India (solid symbols) and the USA (open symbols). Left panel: health-unrelated negative attributions to a stranger increase with the observer’s pathogen disgust sensitivity (subdivided, for clarity of presentation, in intervals of 10 percentiles each). Middle and right panels: health-unrelated negative (middle) and positive (right) attributions to a stranger depend on the observer’s current health conditions. ‘Healthier’: all participants whose self-reported health was ‘very good’ (which turned out to be the bottom 19% of the variable ‘Illness’, *N* = 691). ‘Sicker’: all participants whose health was either ‘average’, ‘fairly poor’, ‘poor’, or ‘very poor’ (which turned out to be the top 19% of the variable ‘Illness’, *N* = 705). Error bars indicate one standard error around the mean.

In general, the findings I have presented here are hard to explain by resorting to a generic halo effect—the tendency for an impression formed in one area to affect opinion in others [17]. Suppose that a face that looks more (less) likeable or intelligent also looks healthier (sicker) as a meaningless consequence, just because positive attributes tend to bunch together and negative ones do too. Unless it is supplemented by entirely ad hoc assumptions, this notion does not explain why the degree of perceived sickliness–healthiness correlates with positive traits (beta = 0.39) far more strongly than with negative ones (beta = –0.13). It does not explain why, in the USA and India, should we find parallel positive-halo effects (Fig. 1, left panel) but wildly diverging negative-halo ones (Fig. 1, right panel). Neither can it account for the role of participants’ own state of health, disgust sensitivity, and country—all of which influence first impressions irrespective of the face’s perceived health and thus of any halo effect involving the latter. Indeed, being in poorer physical conditions was associated with a less favorable overall image of the stranger (Fig. 2, middle and right panels). Being more prone to disgust worsened negative first impressions (Fig. 2, left panel). And living in pathogen-rich India, rather than in the USA, both increased the stranger’s apparent liabilities (Fig. 2, left panel: solid symbols sit higher than open ones) and reduced the power of health cues to allay them (Fig. 1, right panel: the slope is shallower for solid symbols than for open ones). Note that living in a hot and humid country impacts the likelihood that a random stranger is actually carrying pathogens [6, 18]; and one’s pathogen disgust sensitivity and current health conditions reflect the extent of one’s unconscious motivation to avoid infections (see [19–24]). The complementary roles of the stranger’s facial health on one side, and of the observer’s illness, country, and disgust sensitivity on the other converge in suggesting that first impressions of a new face are *adaptively* affected by our concern with infectious disease.

## 4. BAD AND GOOD FIRST IMPRESSIONS MAY REFLECT THE EXPECTED COSTS AND BENEFITS OF INTERACTION

It has been argued that first impressions of a new face reflect an overgeneralization of the typical qualities of people who have a similar face [25]. For example, especially when seeking a mate, it is clearly adaptive to avoid the category of individuals with genetic defects. Such individuals tend to look anomalous, so this repulsion may ‘over’-generalize to unattractive people who are genetically, in fact, perfectly all right. And because individuals with genetic shortcomings tend to be characterized by low power and low competence [26], unattractive people would seem, at first sight, endowed with little power and little competence by association.

In the same vein, one could contend that our adaptive aversion toward sick individuals overgeneralizes to people who only look sick but are actually healthy. This line of thought may predict that sick-looking people take on the distinctive traits of the truly sick—and appear, say, withdrawn, sad, or less energetic. Yet the data presented here show that sicker-looking people are seen as less likeable, less capable, less intelligent, and less trustworthy. With the possible exception of capability, these do not seem to be marks of sickness or even qualities that generically characterize sicker people. Hence, the idea that we are mistakenly transferring attributes from one category of people to another does not appear to be a forceful explanation of the findings presented here.

Contact with strangers bears risks and advantages. Risks may be linked to strangers’ intentions, yet they exist at all times simply because others carry pathogens. And unfamiliar others tend to carry unfamiliar pathogens, which—whether because we have not coevolved with them or have not developed immune defenses against them—are likely to be more dangerous [12, 27]. Advantages comprise opportunities for all manner of social alliances, including marriage and exchange of knowledge and material goods [27]. I propose that negative first impressions of a new face convey the interaction’s expected costs (hence reflect avoidance motivations), and positive ones convey the interaction’s expected benefits (hence reflect approach motivations). Cues of sickliness adaptively alter the tradeoff between benefits and costs, and global first impressions with it.

Note that it hardly matters how, exactly, the study’s participants interpreted the question ‘Does this man look ill or healthy?’: whether, to them, ‘ill’ meant feverish or diabetic or depressed or overweight, and whether this meaning was the same to Indians and to Americans, to the sick and to the well, to the more and to the less easily disgusted. The argument does not change, as our disease-avoidance adaptations steer us away from ailments, deformities, and disabilities of every description, even when we are perfectly aware they are not contagious [28].

Indeed, many such anomalies are utterly unrelated to infectious disease and yet evocative enough to feed automatic social prejudice [29–33]. This is the case for birthmarks [34], obesity [35, 36], physical unattractiveness [37], old age [38], homosexuality [39], mental illness [40], and all manner of physical and behavioral deviations from what is ‘normal’ to us. I have indeed argued [12] that we animals have evolved to use others’ ‘outgroupness’ (a property to which we happen to be exquisitely sensitive) to infer their infectiousness (a volatile, unpredictable, basically unobservable state). Whether we classify a conspecific as ingroup or outgroup is bound to vary in space and time, as these categories are built upon the looks of the individuals to whom (hence, to whose pathogens) we are habitually exposed. Outsiders are likelier to carry pathogens that are novel to us and thus more ominous. Employing outgroupness as a shortcut for infectiousness, then, permits us to continually adjust our defenses to capricious circumstances.

Strangers who look more similar to the people in the local community appear healthier [12]. The analyses reported here indicate that similarity to locals has a further positive effect, not mediated by apparent health, on strangers’ perceived assets—that is, on the likely benefits of interacting with them (see also [41]). The impact of familiar-looking traits on positive attributions held separately for Indian and American observers, for dark- and light-skinned faces, for faces with and without a pathogen cue (see Supplementary Materials for analyses on these subsamples). This effect is consistent with a finding that proves rather alarming in times of pandemics such as COVID-19: people who show glaring signs of infection but look like members of our own group feel more comfortable to be around than people who appear healthy but look very different from us [12]. Yet, of course, the evolutionary function of such an unfair favoritism is clear: even if we have not personally met them before, sick members of our own group ought to be assisted rather than ostracized (a behavior observed in other species too [42]; see [43] for a discussion of the returns of caregiving vs. social distancing in the animal kingdom). Both comfort with contact and a positive first impression help us overcome our instinct to steer clear of the sick.

Facial similarity to locals increased the stranger’s perceived assets but had only a negligible impact on his liabilities. That is, it reduced the expected costs of interacting with him only inasmuch as it made his face look healthier, offering no further returns beyond that. This agrees with the immunologically grounded notion that strangers represent a stable threat. The risks of contact never go away, because anyone can be a source of infection. Such risks simply increase when one’s vulnerability to pathogens does: that is, in people who live in regions of the world richer in pathogens, in people who are more disgusted by sources of contamination, and in people who are in poorer health. These points are revealed by the dependence of a new face’s apparent liabilities on participants’ country, pathogen disgust, and current physical condition. Negative first impressions diminish if the stranger looks healthier, too. Yet, this appears to count little in regions of the world where most fellow humans are likely to be carrying pathogens however ‘healthy’ (i.e. normal) they look.

First impressions are assumed to rely on three fundamental dimensions: attractiveness, trustworthiness, and dominance [4]. Various genetic disorders that end up compromising one’s prospects in life result in facial anomalies: disproportions, asymmetries, oddly shaped eyes or ears or noses or skulls (see [44]). Over evolutionary time, individuals who carried these signs turned out to be less ideal sexual and social mates than individuals who did not. A generalized, better-safe-than-sorry aversion to deviations from the ‘normal’ face (i.e., a lower attractiveness of these faces: see also [26]) is thus exceptionally likely to have evolved. Impressions of attractiveness, in short, may serve to assess others’ suitability as interaction partners. Impressions of trustworthiness may serve to figure out others’ good or bad intentions; impressions of dominance,others’ ability to put intentions into practice [45]. Yet no matter how attractive, trustworthy, or submissive they are, others remain capable of harming us—even killing us—if they are infectious. It makes a whole lot of sense that this vital concern helps shape our first impressions of them.

## Data Availability

The Supplementary Materials (data, analysis scripts, annotated outputs, and a summary table of all results) are publicly available via the Open Science Framework and can be found at https://osf.io/fvce2. The data originally posted by F. van Leeuwen and M.B. Petersen can be accessed at https://osf.io/md7nb.
